# Antioxidative, anticancer, and antibacterial activities of a nanogel containing *Mentha spicata* L. essential oil and electrospun nanofibers of polycaprolactone-hydroxypropyl methylcellulose

**DOI:** 10.1186/s12906-022-03741-8

**Published:** 2022-10-07

**Authors:** Fatemeh Rasti, Yaser Yousefpoor, Abbas Abdollahi, Mojdeh Safari, Ghazaal Roozitalab, Mahmoud Osanloo

**Affiliations:** 1 Noncommunicable Diseases Research Center, Fasa University of Medical Sciences, Fasa, Iran; 2 Student Research Center Committee, Fasa University of Medical Sciences, Fasa, Iran; 3 Department of Medical Biotechnology, School of Paramedical Sciences, Torbat Heydariyeh University of Medical Sciences, Torbat Heydariyeh, Iran; 4 Khalil Abad Health Center, Mashhad University of Medical Sciences, Mashhad, Iran; 5 Department of Microbiology, School of Medicine, Fasa University of Medical Sciences, Fasa, Iran; 6 Department of Medical Nanotechnology, School of Advanced Technologies in Medicine, Tehran University of Medical Science, Tehran, Iran; 7 Department of Medical Nanotechnology, School of Advanced Technologies in Medicine, Fasa University of Medical Sciences, Fasa, Iran

**Keywords:** Nanogel, Antibacterial, Skin cancer, Melanoma, Electrospinning

## Abstract

**Background:**

As the largest organ, the skin has been frequently affected by trauma, chemical materials, toxins, bacterial pathogens, and free radicals. Recently, many attempts have been made to develop natural nanogels that, besides hydrating the skin, could also be used as antioxidant or antibacterial agents.

**Methods:**

In this study, the chemical composition of the *Mentha spicata* essential oil was first investigated using GC–MS analysis. Its nanoemulsion-based nanogel was then investigated; successful loading of the essential oil in the nanogel was confirmed using FTIR analysis. Besides, nanogel’s antioxidative, anticancer, and antibacterial activities were investigated.

**Results:**

Carvone (37.1%), limonene (28.5%), borneol (3.9%), β-pinene (3.3%), and pulegone (3.3%) were identified as five major compounds in the essential oil. By adding carboxymethylcellulose (3.5% w/v) to the optimal nanoemulsion containing the essential oil (droplet size of 196 ± 8 nm), it was gelified. The viscosity was fully fitted with a common non-Newtonian viscosity regression, the Carreau-Yasuda model. The antioxidant effect of the nanogel was significantly more potent than the essential oil (*P* < 0.001) at all examined concentrations (62.5–1000 µg/mL). Furthermore, the potency of the nanogel with an IC_50_ value of 55.0 µg/mL was substantially more (*P* < 0.001) than the essential oil (997.4 µg/mL). Also, the growth of *Staphylococcus aureus* and *Escherichia coli* after treatment with 1000 µg/mL nanogel was about 50% decreased compared to the control group. Besides, the prepared electrospun polycaprolactone-hydroxypropyl methylcellulose nanofibers mat with no cytotoxic, antioxidant, or antibacterial effects was proposed as lesion dressing after treatment with the nanogel. High potency, natural ingredients, and straightforward preparation are advantages of the prepared nanogel. Therefore, it could be considered for further consideration in vivo studies.

## Introduction

Skin, the body's largest organ, has been affected by mechanical, thermal, and physical injury, hazardous substances, damaging UV sunlight, and many pathogens [1]. For instance, skin cancer is the fifth most common cancer worldwide. It is divided into melanoma and non-melanoma [2]. Melanoma is the most aggressive type of skin cancer and accounts for 90% of all skin cancer mortality [3, 4]. Besides, *Escherichia coli* and *Staphylococcus aureus* are two bacterial pathogens that can cause life-threatening infections [5]. *S. aureus* is a gram-positive bacterium mainly responsible for post-operative wound infection, toxic shock syndrome, and food poisoning. *E. coli* is a gram-negative bacterium that lives in the human intestine, and it is the cause of urinary tract disease and wound infections [6, 7].

Drug resistance and side effects of chemical medicine have led to many attempts to develop natural drugs, especially using essential oils (EOs) [8, 9]. For instance, *Mentha spicata* EO possesses many biological effects, including cholinesterase inhibitors, pancreatic lipase inhibitors, antimicrobial, and antiproliferative agents [10–12]. However, for practical application, the potencies of EOs should be improved and prepared in the appropriate dosage form. Preparing nanoemulsions containing EOs is a promising approach to meeting the challenges [13, 14]. Nanoemulsions are uniform dispersion of at least two immiscible liquids together by emulsifier(s) in the nanoscale [15]. Nanoemulsion as a drug delivery system increases the EO's effectiveness and delivery [16]. Furthermore, if the nanoemulsion is gelified, its topical application is facilitated as the viscosity increases [17, 18].

On the other hand, the lesions should be covered after topically administrated dosage forms such as nanogel. Electrospun nanofibers scaffold could be used as a wound dressing; they possess many characteristics such as high surface area, flexibility, and mechanical performance [19]. Besides, Their pores are so small that they prevent the entry of environmental pathogens but do not prevent air exchange with the lesion [20].

To the authors' best knowledge, nanogel containing *M. spicata* EO was not reported. Therefore, in this study, the chemical compositions of the *M. spicata* EO were first investigated. Biological effects, including antioxidant, anticancer, and antibacterial, were then investigated. After that, an attempt was made to improve its efficacy by preparing nanoemulsion-based nanogel. Finally, an electrospun nanofibers scaffold was proposed for after-treatment with the nanogel.

## Materials and methods

### Materials

*M. spicata* EO was purchased from Pharmaceutical Company Essential Oil Dr. Soleimani, Gorgan (36.8418° N, 54.4334° E), Iran. The EO was extracted from bark using the hydro distillation process. Human melanoma cells A-375 (ATCC CRL-1619), *S. aureus* (ATCC 25,923), and *E. coli* (ATCC 25,922) were provided by the Pasteur Institute of Iran. 3-(4,5-dimethyl-thiazol-2-yl)-2,5- diphenyl tetrazolium bromide (MTT) and phosphate-buffered saline (PBS) tablets and, Polycaprolactone (PCL) were provided by Sigma-Aldrich (USA). Hydroxypropyl methyl (HPMC) cellulose was prepared from Nikita, India. Penicillin–streptomycin, trypsin, Dimethyl Sulfoxide (DMSO), and Dulbecco's Modified Eagle's Media (DMEM) cell culture medium were supplied by Shellmax (China). Gibco (USA) produced fetal bovine serum (FBS).

### GC–MS analysis

*M. spicata* EO was analyzed by a gas chromatography–mass spectrometry device (GC–MS) (Alginate 6890, USA) with an HP-5MS silica fused column that connected to a mass spectrometer (Alginate 5973, USA) as described in our previous report [21].

### Preparation and characterization of nanoemulsion-based nanogel

A fixed amount of *M. spicata* EO (10 µL) was mixed with different amounts of tween 20 (10–30 µL) for 5 min at 2000 rpm, room temperature. The PBS solution as an aqueous phase was then added dropwise up to the final volume (5000 µL) and stirred for 40 min. Using a scatteroscope instrument (K-one LTD, Korea) dynamic light scattering (DLS) technique, the nanoemulsion's mean droplet size and distribution were measured as D_50_ and SPAN. SPAN was calculated by D90-D10/D50, where D is the diameter of droplets, and D10, D50, and D90 are the percentile of droplets with a diameter lower than these values. Finally, an optimal nanoemulsion with proper size characteristics, including mean droplet size < 200 nm and SPAN < 1 [22], was selected for further gelation.

The optimum nanoemulsion was gelified by adding carboxymethylcellulose (3.5% w/v); the mixture was stirred overnight in a mild condition (200 rpm, room temperature). The viscosity of nanogel was investigated utilizing a Rheometer machine (MCR-302 model, Anton Pear Co, Austria) in shear rates of 0.1 to 100 S^−1^. Noteworthy, a blank gel was also prepared similarly, only without EO. Furthermore, the nanogel was stored at 4 °C and room temperature for six months and checked for any biphasic or creaming.

### Evaluation of antioxidant activity

Antioxidative activities of *M. spicata* EO and the nanogel were studied by the DPPH test; their serial dilutions (62.5–1000 µg/mL) were prepared using PBS solution containing 0.5% DMSO as the solvent. The assay was performed as follows; 150 μL of DPPH solution (0.3 mM) was first added to each well, and 50 μL of serial dilution was then added. The treated plates were incubated in darkness for 30 min to complete the reaction. The wells' optical density (OD) was read at 517 nm using a plate reader device (Synergy HTX multi-mode, USA). The percentage of antioxidant activity was calculated using Eq. 1.

1 $$\mathrm{Antioxidant effect }\left(\mathrm{\% }\right)=\left(\mathrm{OD control }-\mathrm{ OD sample}/\mathrm{OD control}\right)\times 100$$

### In-vitro cell viability studies

MTT assay was performed to investigate the anticancer activities of the EO and nanogel against A-375 melanoma cells, as described in our previous study [23]. Serial dilutions of the EO and nanogel were prepared in PBS solution containing 0.5% DMSO. Fifty μL cell (1 × 10^4^) was added to each well and incubated for 24 h (37 °C and 5% carbon dioxide) for attached cells and reached a confluence of ~ 80%. The liquid content of each well was then replaced with 50 and 50 μL of fresh complete media culture (RPMI containing 15% FBS and 1% penicillin/streptomycin) and serial dilution and incubated for 24 h. After that, the liquid content of each well was replaced with 50 μL of 0.5 g/ml MTT solution and incubated for 4 h. The formazan crystals were dissolved by adding 50 μL of DMSO to each well, and the OD of wells was read at 570 nm using the plate reader. The cell viability at each concentration was calculated using Eq. (2). Six well/plates were considered control groups filled with PBS solution containing 0.5% DMSO instead of serial dilution.

2 $$\mathrm{Cell\;viability\;\% }=\left(\mathrm{OD\;sample}/\mathrm{OD\;control}\right)\times 100$$

### Evaluation of antibacterial activity

The antibacterial activity of the EO and the nanogel was investigated using the 96-well microdilution test on *E. coli* and *S. aureus* [24]. Serial dilutions (62.5–1000 µg/mL) were prepared using PBS solution containing 0.5% DMSO. New cultured bacterial (24 h) were suspended in the Muller Hinton broth to reach 0.5 McFarland turbidity (1.5 × 10^8^ CFU/ml). Fifty and fifty µL of the bacterial suspensions and PBS solution containing 0.5% DMSO were added to each well. After that, treated plates were incubated at 37 ºC for 24 h, and the OD of wells was read at 630 nm using the plate reader. The percentage of bacteria growth was calculated using Eq. 3. Six/well plates were considered control groups; they were filled with 50 and 50 µL of the bacteria suspension and PBS solution containing 0.5% DMSO.

3 $$\mathrm{Bacteria\;growth }\left(\mathrm{\%}\right)=\left(\mathrm{OD\;sample}/\mathrm{OD\;control}\right)\times 100$$

### Preparation and characterization of nanofibers

PCL-HPMC nanofibers as dressing after treatment with the nanogel were prepared using the electrospinning technique. PCL (12%) and HPMC (3%) polymers were dissolved in HFIP; stirred at 2000 rpm overnight at room temperature. The polymer solution was filled into a 10 mL syringe (with an 18 gauge needle) and placed in an electrospinning machine (Fanavaran Nano-Meghyas, FNM Co, ltd, Iran). Nanofibers were made by optimizing the injection rate (0.6–1.2 mL/h), applied voltage (15–20 kV), and the distance between the needle and the collector (100 rpm, 70–100 mm). A thin layer of aluminum was wrapped around the collector for easy separation of the nanofiber, and it was rotated at 100 rpm. The prepared nanofibers were cut in 0.5 cm and used as an independent sample in the mentioned assays. The wells were filled with 50 µL of bacteria (or cells in MTT tets) suspension, 50 µL of PBS solution containing 0.5% DMSO, and a piece of nanofiber.

A scanning electronic microscopy instrument (SEM) (TESCAN-Vega model, TESCAN Co, Czech Republic) was used to characterize the morphology and size of the nanofibers. Nanofibers mat was cut 0.5 cm and covered with gold vapors by sputtering coating (Q15R-ES model, Quorum Technologies Co, England) before subjecting to SEM devise. Besides, the nanofibers' functional groups and molecular interactions were evaluated by Fourier Transform Infrared (FTIR). For this purpose, PCL, HPMC polymers, and PCL-HPMC nanofibers spectra were obtained using FTIR spectroscopy (Tensor II model, Bruker Co, Germany). Also, the nanofibers' surface hydrophobicity was studied using a contact angle apparatus (CA 500 A model, Sharif Solar Co, Iran). Seven µL of deionized water was dropped on the surface of the nanofibers mat, and its angle with the surface was measured using the apparatus’ software.

### Statistical analysis

All tests were done in triplicates, and data have been reported as mean ± SD; excel software (Microsoft Office, v 2010, USA) was used to calculate means and standard deviations. The independent sample T-test was used to compare different groups with a minimum significance of 0.05 (STATA 16 app, USA). The calculation of IC_50_ was performed using CalcuSyn; v 2011 software (Biosoft England, UK). The lack of overlap between the upper and lower limits of IC_50_ was considered a significant sign.

## Results

### GC–MS analysis of M. spicata EO

The identified components with higher portions than 1% in *M. spicata* EO with GC–MS are listed in Table 1. The five major compounds with 37.1, 28.5, 3.9, 3.3, and 3.3% portions were carvone, limonene, borneol, β-pinene, and pulegone.

**Table 1:** Identified components (> 1%) in *M. spicata* EO by GC–MS analysis

Retention Time	Compound	Area	%	Retention Index
9.4	α-pinene	82,848,781	2.5	932
10.0	camphene	44,325,854	1.3	954
11.1	sabinene	63,810,507	1.9	975
11.2	β-pinene	110,567,647	3.3	979
13.9	*trans*-isolimonene	31,972,473	1.0	984
20.1	limonene	936,146,024	28.5	1029
21.3	borneol	130,589,576	3.9	1169
21.5	α-terpineol	66,211,461	2.0	1188
21.6	dihydrocarveol neo	88,471,507	2.6	1194
23.5	pulegone	108,555,599	3.3	1237
24.1	carvone	1,221,316,667	37.1	1243
28.0	piperitenone	87,099,203	2.6	1343
31.2	*trans*-caryophyllene	34,507,802	1.0	1419

### Physicochemical properties of the nanofibers

**Fig. 1:**
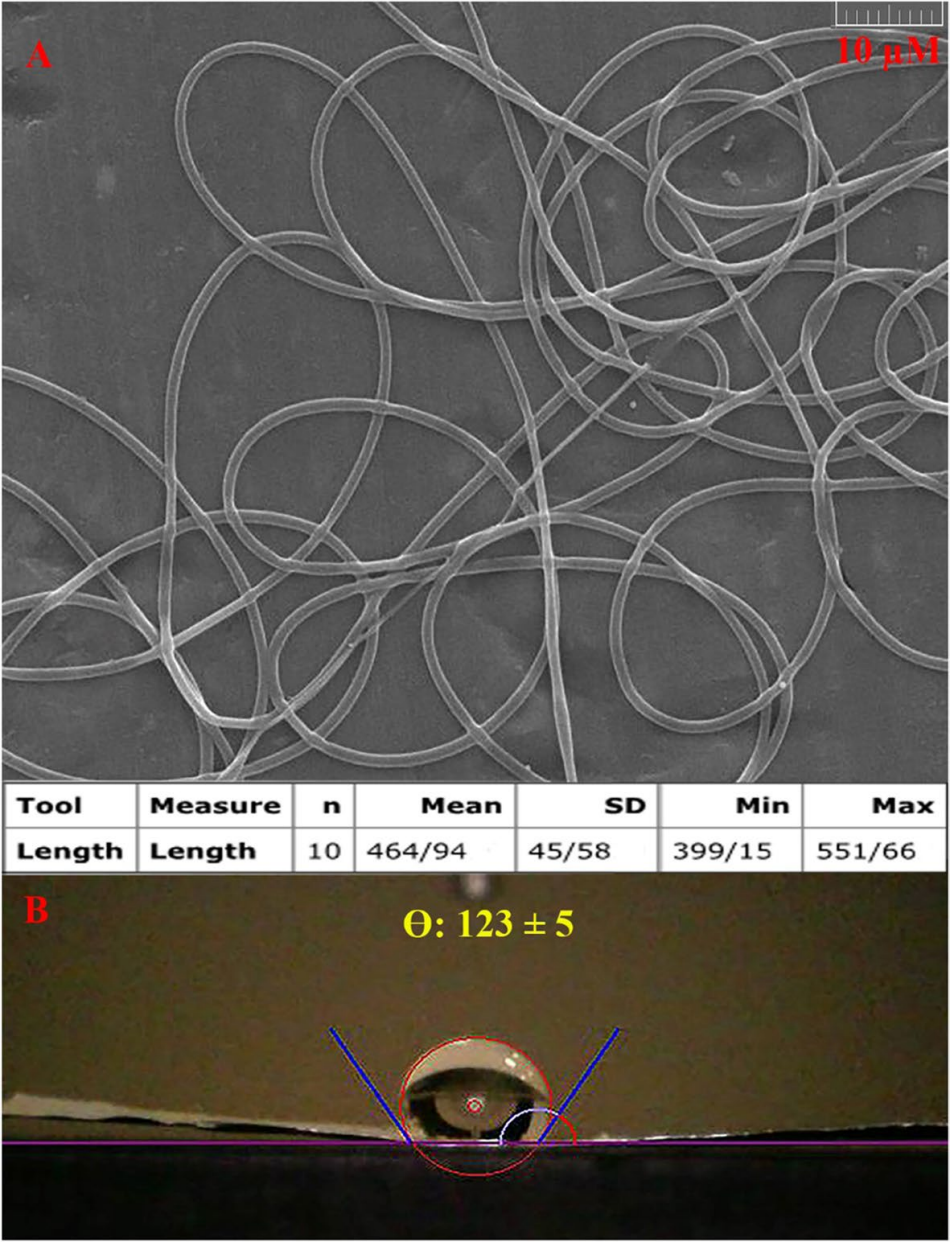
**A** SEM image of polycaprolactone-hydroxypropyl methylcellulose (PCL-HPMC) nanofibers, **B** contact angle of water with surface of nanofibers

The preparation steps for obtaining PCL-HPMC electrospun nanofibers are summarized in Table 2. The nanofibers were obtained at an injection rate of 1.2 mL/h, a distance of 80 mm between the needle and collector, and a voltage of 15 kV. The nanofibers were smooth and randomly oriented with no bead (Fig. 1A). The mean diameter of the nanofibers was 464 ± 45 nm. Moreover, the contact angle of the water with its mat surface was 123 ± 5º (Fig. 1B). It is confirmed to have a hydrophobic surface.

**Table 2:** The steps for optimizing electrospinning parameters for obtaining PCL-HPMC nanofibers

Number	Injection rate (mL/h)	Distance (mm)	Voltage (kV)	Shape result
1	0.6	100	20	Droplet
2	0.7	100	15	Droplet
3	0.8	100	15	Droplet
4	1	100	15	Droplet
5	1.1	80	15	Droplet
6	1.2	70	15	Droplet
7	1.2	80	15	nanofibers

**Fig. 2:**
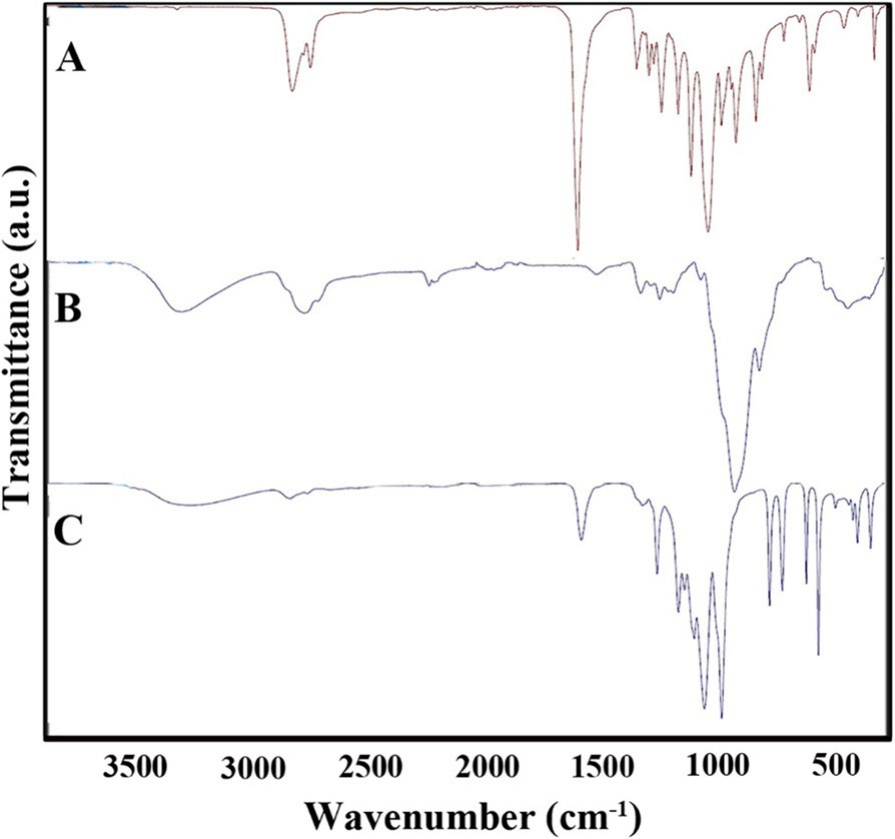
ATR-FTIR analyses of **A**: polycaprolactone (PCL), **B**: hydroxypropyl methylcellulose (HPMC), **C**: PCL-HPMC nanofibers

FTIR spectra of the PCL and HPMC powders and electrospun PCL-HPMC nanofibers are shown in Fig. 2. In the spectrum of the HPMC, the small peak at 2896 cm^−1^ is attributed to the stretching vibration of C-H. The bands that appeared at 1051 cm^−1^ and 3418 cm^−1^ are related to the stretching vibration of C-O, and O–H groups, respectively. Besides, the FTIR spectrum of PCL powder showed an absorption peak at 1722 cm^−1^, which is assigned to the stretching vibration of C = O. The peaks that appeared at 2943 and 2865 cm^−1^ are attributed to the stretching vibration of -CH_2_- [25]. The peak at 1292 cm^−1^ is related to C–C stretching vibration. Another characteristic band at 1236 and 1164 cm^−1^ is assigned to C–O–C symmetric and asymmetric stretching vibrations [26]. In the FTIR spectra of electrospun PCL-HPMC nanofibers, the changes in shape, intensity, and wavelength of some absorption peaks were observed, corresponding to the interaction between PCL and HPMC. For instance, the absorption peak intensity at 1722 cm^−1^ attributed to C = O in PCL powder was changed and shifted to 1702 cm^−1^ in the spectrum of prepared nanofibers. Moreover, when PCL was blended with HPMC, the characteristic peaks of both polymers appeared in the FTIR spectra of PCL-HPMC nanofibers, thus indicating the presence of both PCL and HPMC in the structure of obtained nanofibers [27].

### Characteristics of the nanoemulsions and the nanogel

**Fig. 3:**
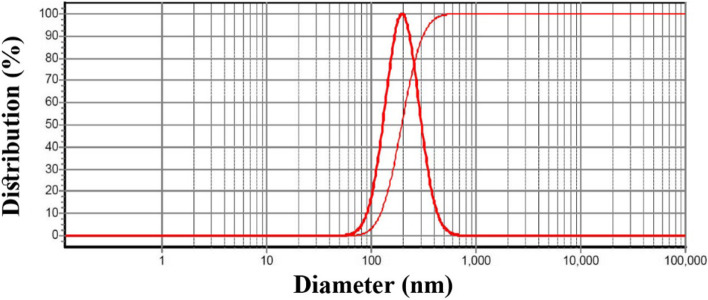
DLS analysis of the nanoemulsion with a droplet size of 196 ± 8 nm and SPAN 0.96

**Fig. 4:**
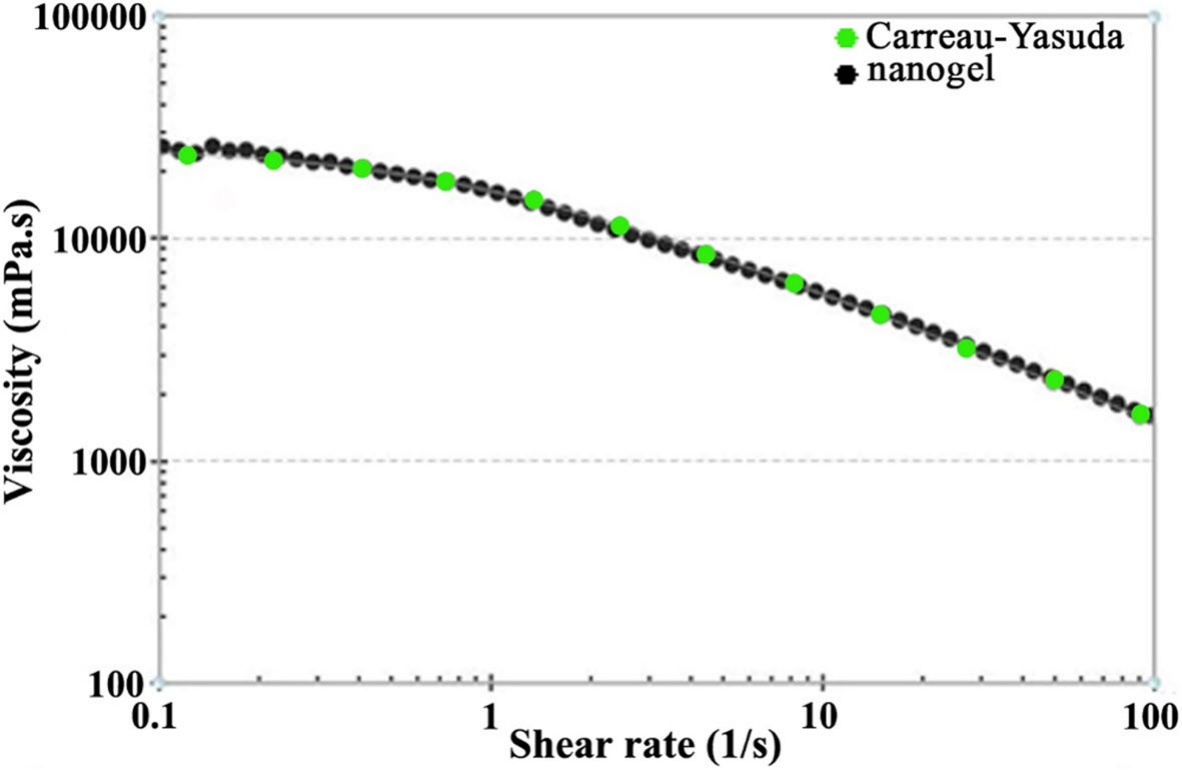
Nanogel viscosity in different shear rates is entirely consistent with Carreau Yasuda regression

Components and size measurements of the prepared nanoemulsions are listed in Table 3. Only NS2 with a mean droplet size of 196 nm and SPAN 0.96 possess proper characteristics; its DLS diagram is depicted in Fig. 3. The nanoemulsion was then gelified; its viscosity in different shear rates is fully fitted with the Carreau-Yasuda model (Fig. 4). It is well-known for non-Newtonian fluids such as polymeric solutions [28]. The viscosity of non-Newtonian decreases with increasing the shear rates and vice versa [29, 30]. Furthermore, no biphasic and creaming was observed in the nanoegel after six months of storage at 4 °C and room temperature.

**Table 3:** Components and characterization of the prepared nanoemulsions

NO	Compound (µL)			Size Analysis	
	***M. spicata* EO**	**tween 20**	**PBS**^**a**^	**particle size (nm)**	**SPAN**^**b**^
NS1	10	10	4980	354	3.48
NS2	10	15	4975	196	0.96
NS3	10	20	4970	189	1.02
NS4	10	25	4965	464	10.83
NS5	10	30	4960	216	8.61

^a^ Phosphate-buffered saline

^b^ droplet size distribution

### Antibacterial properties

**Fig. 5:**
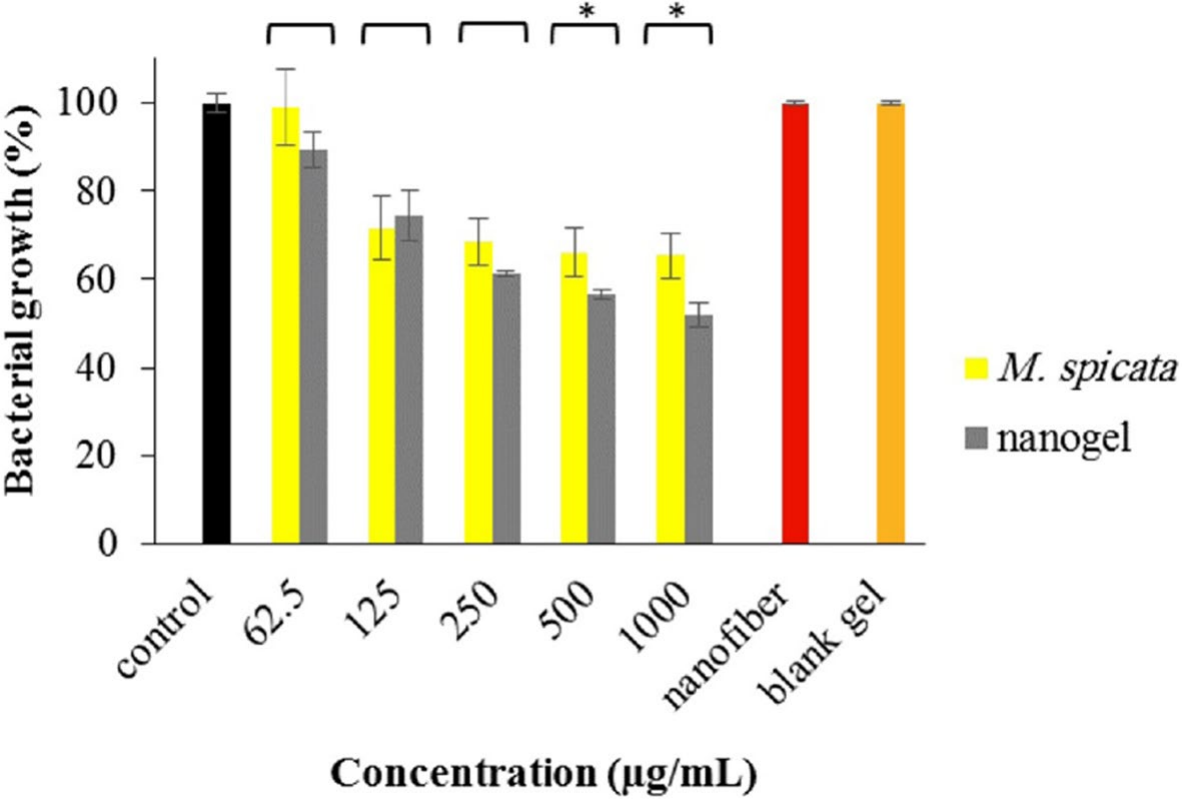
Antibacterial effect of *M. spicata* EO and its nanogel on *E. coli* (*: *p*-value < 0.05)

Figure 5 shows the antibacterial activities of different concentrations of the EO and the nanogel on *E. coli*. The efficacy of the nanogel was significantly more potent than the EO at 500 and 1000 µg/mL; *p*-values = 0.0432 and 0.0156). Interestingly, nanofiber and blank gel did not affect bacterial growth.

**Fig. 6:**
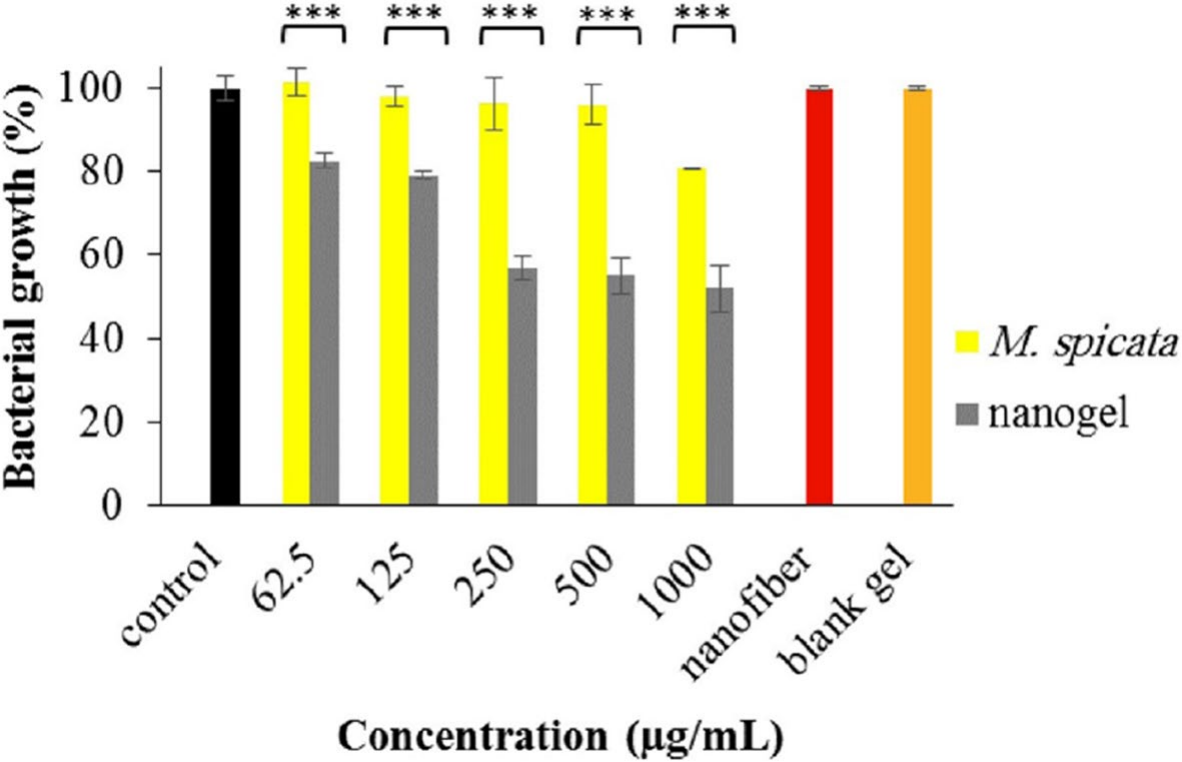
Antibacterial effect of *M. spicata* EO and its nanogel on *S. aureus*. (***: *p-*value < 0.001)

The antibacterial effects of the EO nanogel and blank gel on *S. aureus* are depicted in Fig. 6. Their efficacies are dose-dependent, and there is a significant difference (*p*-value < 0.001) observed between them at all examined concentrations (62.5–1000 µg/mL). The nanofiber and blank gel did not affect the growth of *S. aureus*.

### Antioxidant properties

**Fig. 7:**
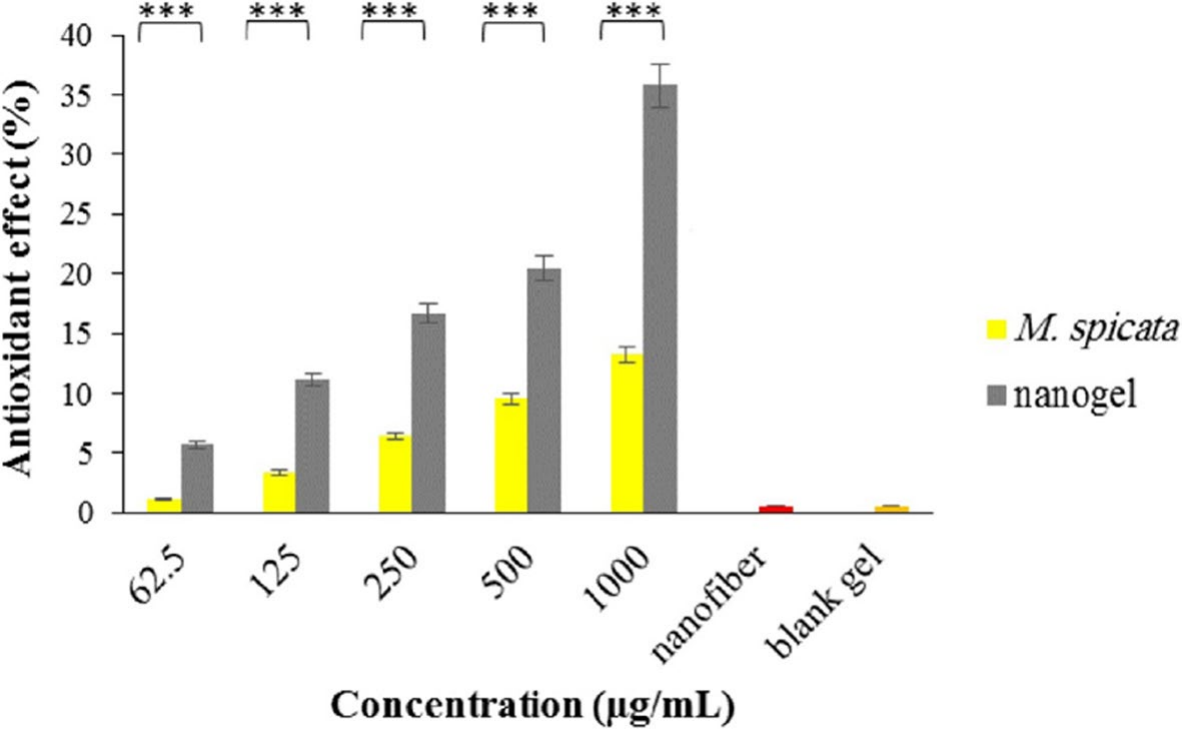
Antioxidative effect of *M. spicata* EO and its nanogel ( ***: *p*-value < 0.001)

Figure 7 shows the antioxidant activities of the EO and the nanogel; the antioxidant effect increases with the increasing concentration. Also, the potency of the nanogel was significantly more potent than the EO (*p*-value < 0.001).

### Anticancer properties

**Fig. 8:**
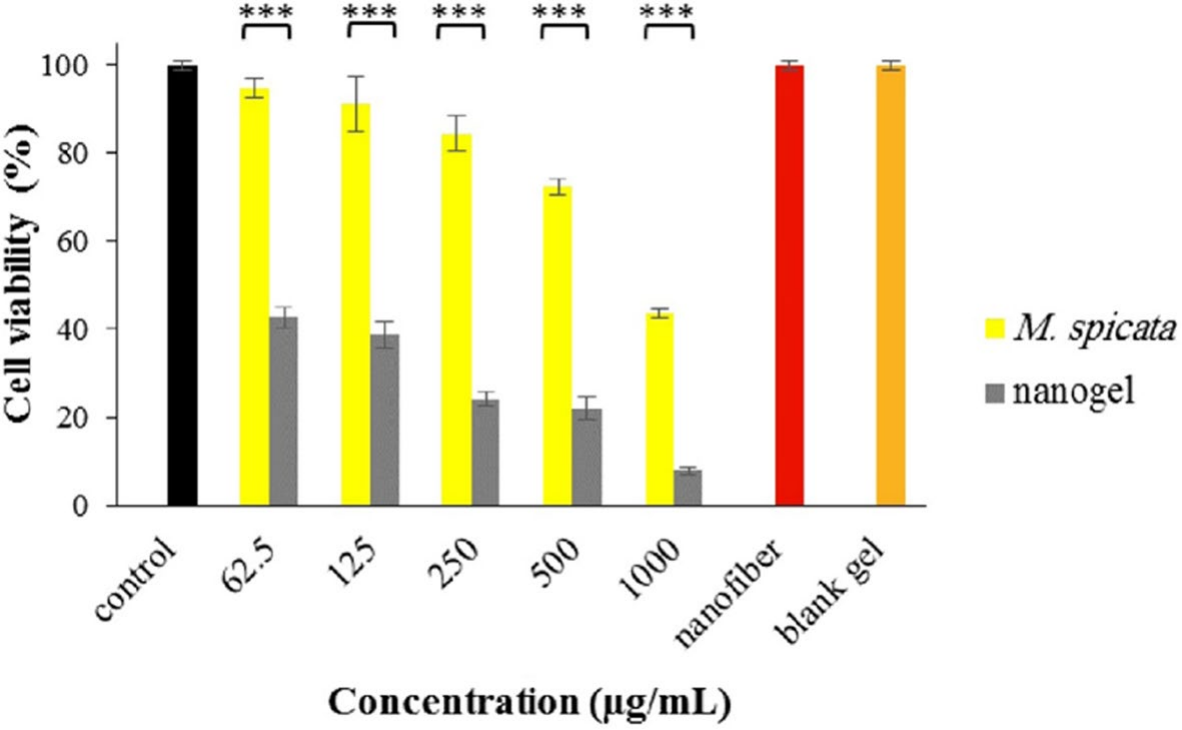
Cytotoxic effect of *M. spicata* EO and its nanogel on A-375 melanoma cell (***: *p-*value < 0.001)

The cytotoxicity effect of the EO and the nanogel A-375 melanoma cells are shown in Fig. 8. The IC_50_ value of the nanogel (55.0 µg/mL) about 18 folds was more potent than the EO with an IC_50_ value of 997.4 µg/mL. Besides, the potency of the nanogel at all concentrations was substantially more than the EO (*p*-value < 0.001). Moreover, the viability of cells 8% was reduced after treatment with blank gel, and nanofiber did not affect the viability of the cells.

## Discussions

In this study, chemical compounds of *M. spicata* EO as a natural medicine were investigated by GC–MS analysis; carvone (37.1%) was the major compound. Previous reports indicated that carvone has antimicrobial, anticonvulsant, antioxidant, and antitumor potentials [31]. The preparation of EO-based nanoformulations as a promising approach for improving the efficacy and designing the appropriate dosage forms (topical, systemic, or inhalation) has recently been more considered [32, 33]. High-energy and low-energy (spontaneous) methods are common in nanoemulsions preparation [34]. This study used the low-energy method to prepare the nanoemulsion to prevent the evaporation of EO’s volatile components. Besides, as the low viscosity of nanoemulsions is a challenge for topical applications, the optimum nanoemulsion was thus gelified in the current study. Interestingly, nanogel containing *M. spicata* EO was not reported so far. Therefore, this study investigated the biological properties (antibacterial, antioxidant, and anticancer properties) of the nanoemulsion-based nanogel containing *M. spicata* EO.

The efficacy of the nanogel was significantly more potent than the EO against *E. coli* and *S. aureu*s. Besides, *M. spicata* EO and nanogel were found to have antioxidant activity; the nanogel was significantly more potent than unformulated EOs. Free radicals are involved in melanoma development, causing adverse skin effects such as aging and inflammation [35, 36].

The cell wall of Gram-negative bacteria is more complex than Gram-positive bacteria and generally is more resistant [37]. However, the efficacy of the EO against *E. coli* was more potent than *S. aureus* in the current study. It is accepted that EO has selective effects on gram-negative or positive bacteria [38, 39]. For instance, *Myrtus communis* EO with IC_50_ 4547 µg/mL on *E. coli* was significantly more potent against *S. aureus* (IC_50_ 394 µg/mL) [33]. Besides, *Mentha piperita* EO with IC_50_ 27 mg/mL on *S. aureus* was significantly more potent against *E. coli*, IC_50_ 18 mg/mL [34].

Furthermore, some reports on nanogels containing EOs from other species in the *Mentha* family have been published. For instance, the growth of *S. aureus* after treatment with 1250 µg/mL nanogel containing *M. piperita* EO 30% decreased [40]. Besides, no significant effect (< 10%) on the growth of *P. aeruginosa* and *S. aureus* was reported after treatment with 2500 µg/mL nanogel containing *M. longifolia* EO [20].

In the current study, the efficacy of the nanogel was 18 folds more potent than the EO against A-375 melanoma cells; IC_50_ values were obtained at 55.0 and 997.4 µg/mL. Cancer cells' membranes are wider than normal cells for obtaining nutrient molecules, and they have downregulated gap junctions and become ready for metastasis [41, 42]. The weak lymphatic system with large gaps are two important factors in inactive nano-drug delivery systems; nanostructures easily enter cancer cells and are not allowed to exit, improving their efficacy [43, 44]. Moreover, large amounts of EO droplets could be loaded into one nanostructure like nanogel, improving efficacy [38, 45].

After topical treatment of nanogel, a dressing is required to cover the lesions. It should prevent pathogens from entering the body and, at the same time, allow air exchange [46]. Nanofibers with a high surface area, small pore size, and high porosity have attracted huge interest in wound dressing in the last decades [47]. Electrospinning is still a promising technique for preparing nanofibers [48]. PCL is an excellent dressing with promising properties, including biocompatibility and slow degradation rate [49–51].

Furthermore, solid surfaces are classified into five categories according to the angle (θ) at which the water drop forms; 0–30° strongly wettable, 30–75° moderately wettable, 75–105° neutrally wettable, 105– < 150° hydrophobe, 150–180° super hydrophobe [20]. The angle of water with the PCL nanofibers was reported at 150° [46]. To improve its hydrophobicity, a hydrophilic polymer is commonly added. For instance, polycaprolactone-alginate electrospun nanofibers with 188 nm diameter and a contact angle of 144° for antibacterial dressing was introduced [20]. Polycaprolactone-chitosan with 200 nm diameter and contact angle of 109° for dressing cutaneous leishmaniasis was proposed [52]. The current study reduced the angle to 123° by adding HPMC. The prepared PCL-HPMC nanofiber (with no-cytotoxic, antibacterial, and antioxidant effects) is proposed as a dressing to cover the treated area.

## Conclusions

This study aimed to improve the biological activities of the *M. spicata* EO by preparation of the nanogel containing the EO. Interestingly, the antibacterial, antioxidant, and anticancer effects of the nanogel were significantly more potent than the EO. Besides, the nanogel could thus be considered for further consideration in vivo studies.

## Data Availability

All data is available from corresponding authors on reasonable request.
